# Pigmented villonodular synovitis of the temporomandibular joint with skull base extension: a retrospective case series

**DOI:** 10.1038/s41598-022-09732-6

**Published:** 2022-04-06

**Authors:** Qiang He, Xin Zan, Fei Chen, Chao You, Jianguo Xu

**Affiliations:** 1 Department of Neurosurgery, West China Hospital, Sichuan University, 37 Guoxue lane, Chengdu, 610041 Sichuan China; 2 Department of Otorhinolaryngology-Head and Neck Surgery, West China Hospital, Sichuan University, 37 Guoxue lane, Chengdu, 610041 Sichuan China

**Keywords:** Head and neck cancer, Cancer therapy

## Abstract

Most studies on pigmented villonodular synovitis (PVNS) of the temporomandibular joint (TMJ) with skull base extension mostly are case report. Here, we summarize the clinical features, treatments, and outcomes of PVNS of the TMJ with skull base extension in a large case series. We reviewed the clinical information relating to patients diagnosed with PVNS of the TMJ with skull base extension information of patients in our center between 2011 and 2020. We reviewed 10 patients (4 males and 6 females). All cases had presented with a unilateral lesion extending the middle skull base. PVNS of the TMJ with skull base extension occurred on the left side in 6 patients (60%) and on the right side in 4 patients (40%). Of the 10 patients, pain and mass were the most prevalent symptoms. All patients received surgery and no recurrence was seen after 35.90 ± 25.35 months follow-up. Despite destructive biological behavior, surgery can achieve an excellent outcome for patients with PVNS of the TMJ with skull base extension. An en bloc resection may prevent recurrence and provide long-term relief. Radiotherapy may be reserved for subtotal excision and recurrent lesions but require further investigation.

## Introduction

Pigmented villonodular synovitis (PVNS), also referred to as a diffuse-type tenosynovial giant cell tumor (D-TGCT), or a giant cell tumor (TGCT) of the tendon sheath, is a rare, proliferative, locally destructive, and mesenchymal neoplasm with a relatively higher recurrence rate^1^. This condition often affects larger joints such as the knee and hip, although other joints may also be affected, such as the ankles and shoulders^2^. Initially considered as an inflammatory disease, cytogenetic studies revealed that PVNS is associated with a chromosomal translocation: t (1; 2) (p13; q35) that fuses *COL6A3* (encoding collagen type VI α3) to colony-stimulating factor 1 (*CSF-1*), and also revealed that PVNS is a neoplasm that can exhibit destructive behavior^3^. The main treatment is resection which can achieve excellent clinical outcomes. Studies have also shown that this disease may be caused by chronic inflammation^4^, although recurrent trauma and hemarthrosis may also be risk factors^5^.

PVNS in the TMJ is extremely rare; the combination of this condition with the extension of the skull base is even rarer. In this present, we reviewed a large series of cases from our hospital to summarize this condition by its clinical features, treatment, and outcomes.

## Materials and methods

The institutional review board of West China Hospital of Sichuan University approved the present study in accordance with the World Medical Association Declaration of Helsinki (2002 version). Written informed consent was obtained from each patient or family member. Patient information was identified by different terminologies, including PVNS, TGCT, and giant cell tumor of the tendon sheath in the TMJ with a skull-extending base. We retrospectively reviewed 10 patients from January 1, 2020, to December 31, 2020. The clinical information, including age, sex, complaint, radiological features, pathological findings, treatment methods, and clinical outcome were recorded.

Preoperative and postoperative contrast-enhanced magnetic resonance imaging (MRI) were scheduled in all patients. The latter was given to evaluate the surgical extent of the tumor. Gross total resection (GTR) was defined that no residual tumor was observed on postoperative MRI, otherwise, subtotal resection (SR) was considered. Patients were performed with hematoxylin–eosin stain (H&E) and immunohistochemical examination. The follow-up interval was 3 months, 6 months, and 1 year after surgery. Contrast enhancement MRI was performed during follow-up. Telephone consultation was constant.

### Ethical approval

All experimental protocols were approved by Ethics Committee on Biomedical Research, West China Hospital of Sichuan University. All methods were carried out in accordance with the relevant guidelines and regulations. Informed consent was obtained from the participant.

## Results

### Patient characteristics

The characteristics of 10 patients were shown in Table 1. Four males and six females were included ranging from 18 to 60 years old with the mean age were 41.80 ± 13.86 years old. Six patients occurred on the left and four patients on the right. All cases presented with a unilateral lesion. The most frequent discomforts were pain (4/10, 40%) and mass (4/10, 40%) with a mean duration of 13.90 ± 12.76 months.

**Table 1:** Baseline characters 10 Patients of PVNS in the TMJ stretching of the skull base.

No	Age	Side	Sex	Nationality	Chief complaint	Duration of Symptoms, mo	Radiotherapy	Operation	Follow up, mo
1	48	L	F	Han	Swelling pain	2	Yes	SE	66
2	47	R	F	Han	Blurred vision, ear pain	6	No	EBR	42
3	48	R	M	Tibetan	Tinnitus, hearing loss	36	No	EBR	8
4	24	R	F	Han	Headache	1	No	ER	3
5	48	L	F	Tibetan	Mass, dizziness, pain	18	No	EBR	11
6	27	L	F	Han	Mass	2	No	EBR	21
7	18	L	M	Han	Mass	24	No	EBR	55
8	60	L	F	Han	Restricted mouth opening	24	No	EBR	47
9	44	L	M	Han	Swelling pain	24	No	EBR	3
10	54	R	M	Han	Mass	2	No	EBR	1

*L* Left, *R* Right, *F* Female, *M* Man, *EBR* en bloc resection, *ER* Extensive resection, *SE* Subtotal excision, mo = month.

### Radiological characteristics

**Figure 1:**
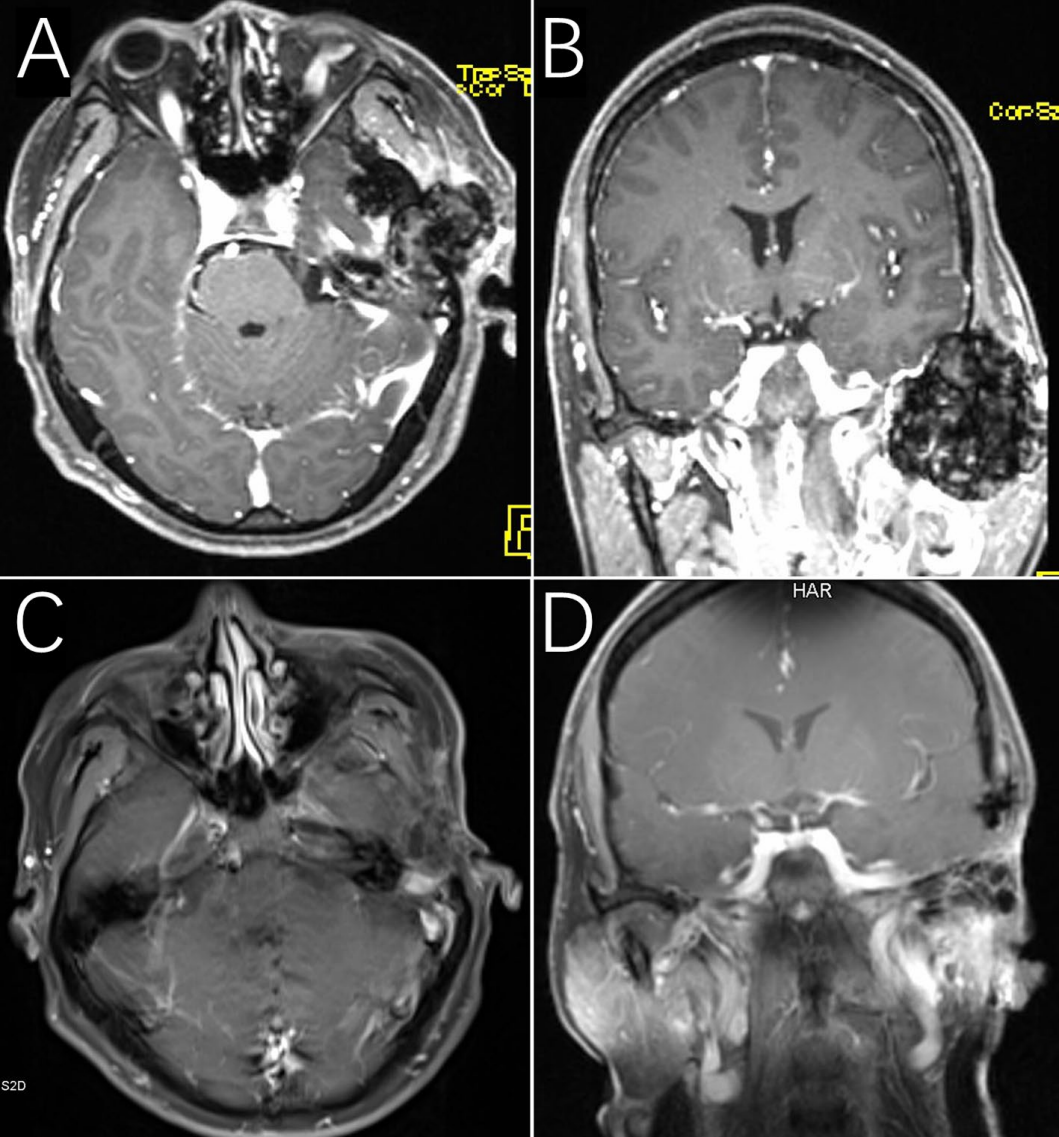
Patient 9: PVNS on MRI showed modest inhomogeneous contrast enhancement, expansive growth and skull base invasion (**A** and **B**). After 8 months of follow-up, the tumor had not recurred (**C** and **D**).

The lesion on MRI presented expansive growth and slight enhancement with skull base invasion associated mass effect on the temporal lobe (Fig. 1A,B). Eight months after surgery, no recurrence of the tumor was observed (Fig. 1C,D).

### Pathological findings

**Figure 2:**
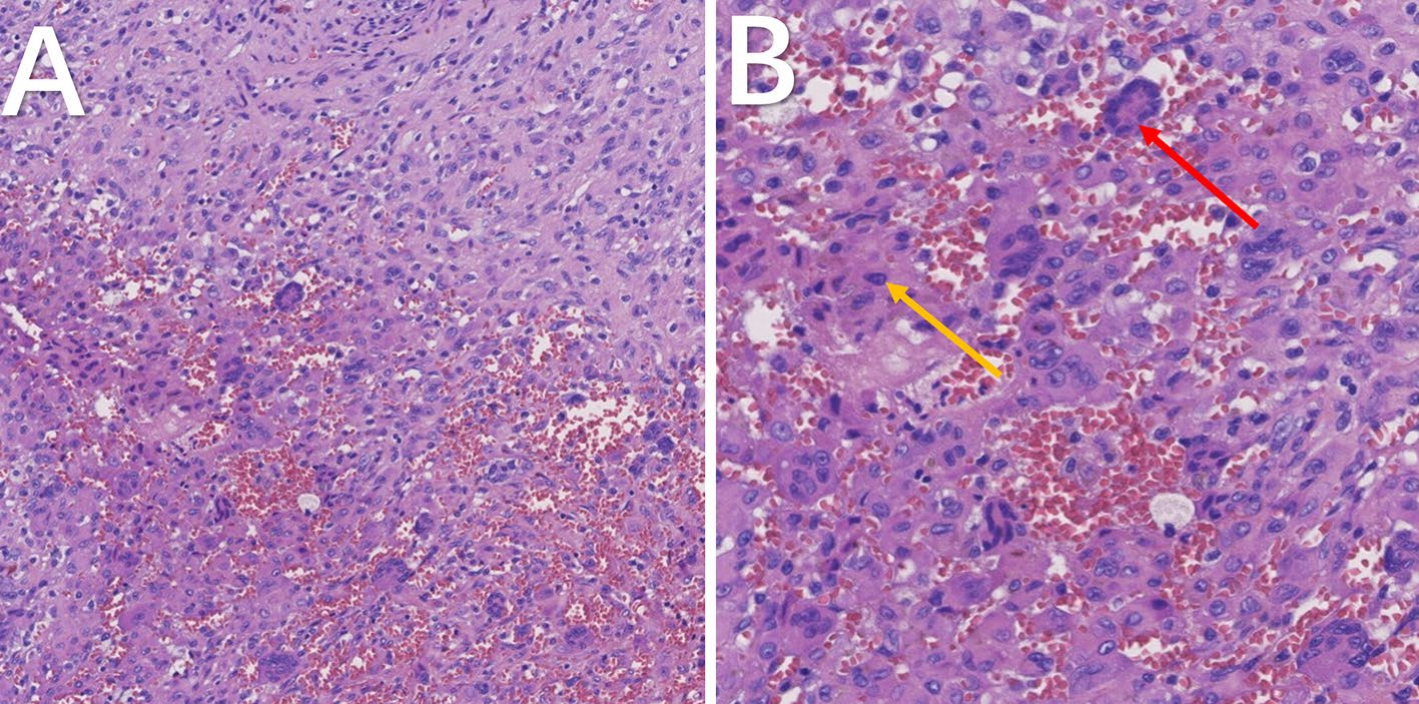
Representative histology from patient 9. The lesion composed of plenty of multinucleated giant cells (red arrow) and mononuclear cells (yellow arrow) with hematoxylin–eosin stain (**A** and **B**).

Microscopically, the H&E findings were presented. The lesion revealed clusters of multinucleated giant cells and adjacent hemosiderin-laden mononuclear cells (Fig. 2A,B).

### Treatment and follow-up

**Figure 3:**
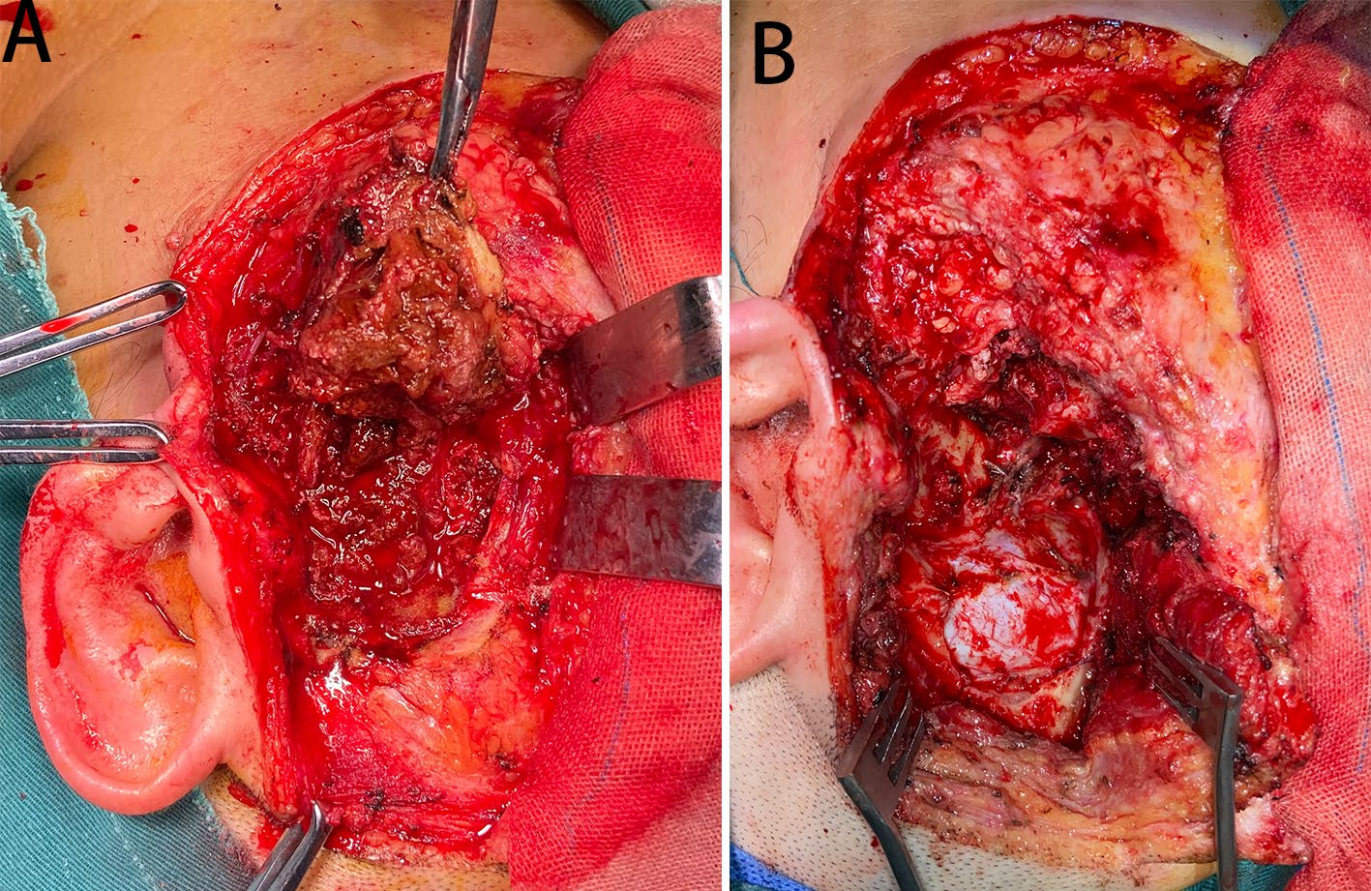
Representative histology from patient 9. Intraoperative photos of tumor resection showed the lesion was removed (**A** and **B**).

All patients received surgical treatment with the preauricular infratemporal fossa approach (Fig. 3A,B, Table 2). Nine patients were performed with GTR, including the tumor and the affected tissues, such as the skull base bone, dura mater, synovium. One patient underwent SR due to facial nerve encapsulation, who then underwent GTR. The patient had facial paralysis after GTR. One patient occurred a surgical site infection and recovered smoothly after we removed the titanium mesh graft for reconstruction. The average follow-up time was 35.90 ± 25.35 months and no recurrence were observed.

**Table 2:** Operative information of 10 Patients of PVNS in the TMJ with the skull base extension.

No	Extent of lesion	Preoperative embolization	Surgeons	Intraoperative finding	Complication
1	SB, TMJ, TS	None	O	Involving MKB	None
2	TMJ, TS	None	NS	3 × 3 × 3 cm mass with rotten fish, bone destruction and abundant blood supply at MKB	Incision infection
3	TB, SB, ZB, EAC, AO	None	O + NS	Lesion with mixed property, dark red and eroding the IAC and MKB	None
4	TB, TMJ	None	NS	Tumor located in the MKB which is solid and has no abundant blood supply	None
5	TB, TMJ	Yes	NS	Lesion involving the whole MKB, infratemporal fossa, maxillofacial region, dura mater and TS	Incision infection
6	TB, TMJ	None	NS	Tough and obscure boundary tumor about 3 × 3.8 × 2.1 cm	None
7	TB, TMJ	None	O	Grayish brown and tough tumor with a size of 3.5 × 4.0 cm and invading the TMJ, MKB	None
8	TB, TMJ, SB, ZB	None	O	Brittle and easily bleeding tumor invading MKB, EAC, and reaching pterygopalatine fossa	None
9	TMJ, EAC, MKB	None	O + NS	Light brown about 5 × 6 cm in size and tough texture	None
10	TB, EAC, MKB	None	O	Invading the right TMJ, tough texture, unclear boundary, and poor blood supply	None

*SB* sphenoid body, *TS* temporal squama, *TB* temporal bone, *ZB* zygomatic bone, *EAC* external auditory canal, *AO* Auditory ossicles, *MKB* middle skull base, *IAC* internal auditory canal, *NS* Neurosurgeon, *O* Otolaryngologist.

## Discussion

PVNS of the TMJ with skull base extension is exceedingly rare. Almost all researches were a case series report and retrospective study. We aimed to present a large case series of the condition.

Contrary to other studies, our study found that the condition had male predominance (1.64:1) which was contrary to other studies^6^. The disease usually occurred in the middle-aged, although it could be seen even at the age of 18 in our case series. Pain and mass were the most common presentations; symptoms related to neurological symptoms such as hearing loss and tinnitus were not uncommon, especially in cases with serious petrosal bone erosion^7^. Trauma has been reported to be contributing factor^5^, but it was not common.

Imaging examinations are necessary for the initial and differential diagnosis, and surgical evaluation^8^. Careful reading images before surgery are important for surgical planning. On MRI, the lesion generally shows low signal intensity on T2 images and “blooming” artifact on gradient-echo images due to the high hemosiderin concentration^9^. The lesion on contrast MRI presents variable levels of enhancement. CT mainly presents bony destruction of the mandibular condyles and skull base invasion and areas of central calcification. The differential diagnosis mainly consists of chondroblastoma, reparative granuloma, chondrosarcoma, giant cell tumor of bone, and synovial sarcoma. Digital subtraction angiography may show highly vascular lesions with multiple arteries blood supply and arteriovenous shunting, especially for giant tumors. It is useful to conduct preoperative embolization to decrease blood loss during operation. One of our cases was performed digital subtraction angiography embolism because the patient had a big tumor with 3.8 cm in diameters.

Pathologic evaluation is essential for a definitive diagnosis. Biopsy and postoperative pathological examination are the most common method. Because PVNS of TMJ is involved in the facial region and the tumor is relatively superficial, the pathologic examination before surgery provides valuable information for diagnosis and treatment. FNAB with the guidance of ultrasound or CT is a simple method^10^. Pathologic results reveal that the lesion is rich in giant cells and focal cartilage formation. Different kinds of cells, including mononuclear histiocytes, multinucleated giant cells, and xanthoma cells, are present in the lesion^11^. The differential diagnosis includes giant cell granuloma, giant cell tumor, and brown tumor^12^. Due to the overlapping of histopathological characteristics of these lesions, the definitive diagnosis may need additional testing tools. A brown tumor can be ruled out with normal parathyroid and calcium levels. Giant cell tumors are generally more destructive. Giant cell granulomas are more commonly bone-based. Evidence of hemosiderin deposition may also aid in the appropriate diagnosis of PVNS^6^. In our cases, one patient has undergone an FNAB test.

The most effective treatment for the condition is surgery. GTR leads to a lower recurrence rate and is associated with a longer period of relief when compared with SR. However, GTR may bring damage to occlusal function because of the destruction of the functions of the TMJ. Some scholars think that SR may be a risk factor for recurrence^13^. RT after SR for residual tumors is an adjuvant treatment^14^. The most common radiation dose is 35–60 Gy in 20–30 fractions. Therefore, GTR should be conducted when it was feasible. When complete removal of the tumor could not be achieved, RT after surgery is recommended. In our series, GTR was achieved in 9 patients and 1 patient had SR for facial nerve preservation. Truly GTR included completely bony resection and soft tissue resection. We often perform extended removal for suspicious bony invasion including the greater sphenoid wing, squamous and petrous parts of the temporal bone. All our cases were extradural lesions, invasion of the periosteal layer of the dura mater was found in 10 patients. Meticulously sharp separation was performed to remove the outer layer dura for complete resection and preserve the inner layer to prevent leakage of CSF. The operation involved multi-disciplinary cooperation, including neurosurgeons, otolaryngologists, and maxillofacial surgeons. Multi-disciplinary cooperative surgeries could achieve more radical resection, reliable skull base reconstruction, and better cosmetic results.

There are two views on defect repair. Safaee et al.^6^ believed that it was not necessary to reconstruct the middle skull base and that autologous fat can be used to fill large defects. However, Liu et al.^15^ reported that repairing bony defects in the skull base can provide a satisfying contour and stable support for the brain. Unfortunately, there is a lack of enough information relating to the comparison of cases with and without reconstruction. We mainly used temporalis muscle flaps and autologous fat to reconstruct the skull base, especially for big tumors with large defects^16^.

Postoperative facial palsy is a common complication after surgery. Therefore, the perseveration of the facial nerve is important. Intraoperative electrophysiological monitoring could identify and protect the facial nerve. Although facial palsy was the most common postoperative complication, it was worth that we should perform GTR because facial palsy could recovery to some extent.

In conclusion, although PVNS of the TMJ with the extension of the skull base is destructive and leads to intracranial extension, surgery can achieve an excellent outcome, especially for GTR. The most common symptom associated with this condition is pain and swelling. RT may be reserved for extensive or recurrent disease. Because of the relatively high rates of recurrence, a sufficient period for follow-up is necessary.
